# Time-varying generalized linear models: characterizing and decoding neuronal dynamics in higher visual areas

**DOI:** 10.3389/fncom.2024.1273053

**Published:** 2024-01-29

**Authors:** Geyu Weng, Kelsey Clark, Amir Akbarian, Behrad Noudoost, Neda Nategh

**Affiliations:** ^1^Department of Biomedical Engineering, University of Utah, Salt Lake City, UT, United States; ^2^Department of Ophthalmology and Visual Sciences, University of Utah, Salt Lake City, UT, United States; ^3^Department of Electrical and Computer Engineering, University of Utah, Salt Lake City, UT, United States

**Keywords:** higher visual areas, time-varying systems, generalized linear model, encoding and decoding, behavioral readout, visual perception

## Abstract

To create a behaviorally relevant representation of the visual world, neurons in higher visual areas exhibit dynamic response changes to account for the time-varying interactions between external (e.g., visual input) and internal (e.g., reward value) factors. The resulting high-dimensional representational space poses challenges for precisely quantifying individual factors’ contributions to the representation and readout of sensory information during a behavior. The widely used point process generalized linear model (GLM) approach provides a powerful framework for a quantitative description of neuronal processing as a function of various sensory and non-sensory inputs (encoding) as well as linking particular response components to particular behaviors (decoding), at the level of single trials and individual neurons. However, most existing variations of GLMs assume the neural systems to be time-invariant, making them inadequate for modeling nonstationary characteristics of neuronal sensitivity in higher visual areas. In this review, we summarize some of the existing GLM variations, with a focus on time-varying extensions. We highlight their applications to understanding neural representations in higher visual areas and decoding transient neuronal sensitivity as well as linking physiology to behavior through manipulation of model components. This time-varying class of statistical models provide valuable insights into the neural basis of various visual behaviors in higher visual areas and hold significant potential for uncovering the fundamental computational principles that govern neuronal processing underlying various behaviors in different regions of the brain.

## 1 Introduction

Understanding how our behavior is generated through the integration of sensory, motor, and cognitive information is a fundamental goal of systems neuroscience. Such an understanding relies heavily on the ability to quantitatively describe the individual and interactive effects of external and internal factors on neuronal responses. Classically, the visual system has been described as comprised of two main parallel pathways responsible for constructing visual perception, both originating from the primary visual cortex (V1) (Newcombe and Russell, 1969; Mishkin et al., 1983; Haxby et al., 1991). The dorsal pathway is dedicated to handling spatial information and extends its projections to the parietal association cortex, often referred to as the “where” pathway. In contrast, the ventral pathway focuses on processing form-related information about objects and directs its projections to the temporal association cortex, commonly known as the “what” pathway [for a more nuanced view, see (de Haan and Cowey, 2011; Zachariou et al., 2014; Sereno et al., 2020)]. Within each pathway, cortical areas are arranged in a hierarchical manner, and visual information undergoes sequential processing increasing in complexity (Desimone et al., 1985; Felleman and Van Essen, 1991). At each stage of the hierarchy, the spatiotemporal sensitivity of the neurons, referred as the neurons’ receptive field, is used to describe the functional properties of the neuron in visual information processing. Classically, the neurons’ receptive field is measured using the linear correlation between simple sensory stimuli controlled by the experimenter and the measured firing rate of the neuron. Although this functional description has generated important insights into sensory processing, the fact that it often assumes the neuron’s spatiotemporal sensitivity remains unchanged over time, resulting in a time-invariant receptive field, provides a limited view about the function of the neuron or the circuit. Indeed, at each stage of the hierarchy, the neuron’s response properties can be modulated by various sensory or non-sensory factors (Meyer et al., 2014). The interactions with these factors may not follow a serial structure (Kandel, 2013). For example, visual areas including V1-V4 and the middle temporal area (MT) also receive top-down feedback from the frontal and temporal lobes (Noudoost et al., 2010; Anderson et al., 2011; Gregoriou et al., 2012; Gilbert and Li, 2013; Kandel, 2013; Markov et al., 2014a,b). These modulations can act at various timescales and in different areas. For example, adaptation to changes in the statistics of the visual environment or the context-dependent sensory processing can happen on a continuum of timescales, from as early as the retina to higher cortical areas.

Understanding the neuronal response dynamics becomes even more challenging in higher visual areas where the modulations can be as fast as milliseconds (ms), with a simultaneous influence of numerous task-related, cognitive, or behavioral factors, interacting both with one another and with incoming sensory signals in possibly a highly nonlinear manner. Examples of such modulatory factors include covert attention (Treue and Maunsell, 1996; Womelsdorf et al., 2006; Mitchell et al., 2007; Anton-Erxleben et al., 2009; Gregoriou et al., 2012), reward (Leon and Shadlen, 1999; Ikeda and Hikosaka, 2003; Ding and Hikosaka, 2006; Kennerley and Wallis, 2009), working memory (Fuster and Alexander, 1971; Fuster, 1990; Merrikhi et al., 2017; Bahmani et al., 2018), salience (Gottlieb et al., 1998; Thompson and Bichot, 2005), expectation (Anderson and Sheinberg, 2008; Puri et al., 2009; Noudoost et al., 2017), task rules (Wallis et al., 2001; Everling et al., 2006; Muhammad et al., 2006; Mante et al., 2013), and motor plans (Chelazzi et al., 1993; Lawrence and Snyder, 2009; Moore and Chang, 2009; Liu et al., 2010). The influence of such modulatory factors can operate on fast timescales and at the level of single trials. This flexibility in the neuron’s encoding of sensory information into spiking activity and in the brain’s decoding of this activity is likely key for behavioral flexibility. A quintessential instance involving fast visual response modulation and changes in our perception of the visual world is saccadic eye movements (saccades), which are rapid eye movements that shift the center of gaze to a new location in the visual field (Schlag and Schlag-Rey, 2002; Marino and Mazer, 2016). Saccade experiments and saccade-related visual behavior stand as a potent and classical paradigm in visual neuroscience, serving as a robust tool to establish links between neural responses and behavior or cognition (Honda, 1989; Nakamura and Colby, 2002; Awater and Lappe, 2004; Jeffries et al., 2007; Zirnsak et al., 2011; Irwin and Robinson, 2015; Wurtz, 2018). Saccade paradigms have been implemented to unravel the intricate physiological and circuit mechanisms underlying various cognitive processes, including visual perception, attention, memory, and more (Deubel and Schneider, 1996; Ross et al., 1997; Friston et al., 2012; Cronin and Irwin, 2018; Edwards et al., 2018; Akbarian et al., 2021).

The complexity and rapid timescale of interaction between sensory and behavioral or motor variables creates a high-dimensional representational space, which can be experimentally intractable, and the experimenter will have to choose a subset of the space for characterization of neuronal sensitivity. Statistical methods can alleviate the subjectivity of this approach by uncovering the nature of computations in terms of mathematical functions underlying the neuron’s response generation (described by an encoding model). However, the high dimensionality of the sensory representation during time-varying behavior makes it challenging to computationally quantify the individual contributions of various factors to neuronal responses with high temporal precision. In addition to challenges for characterizing information encoding, how these factors play a role in generating the instantaneous readout of sensory information can pose a challenge for quantitatively characterizing the information decoding. Therefore, developing unified encoding and decoding frameworks will provide a general tool for establishing links between neurophysiological response modulations and behavioral phenomena, applicable to many different cognitive tasks and brain areas.

The point process generalized linear models (GLMs) provide a powerful framework to quantitatively characterize sensory processing and read out sensory information from neuronal responses at the level of single-trial spike trains and individual neurons (Paninski, 2004; Simoncelli et al., 2004; Meyer et al., 2016). The GLM approach has been widely used to characterize the responses of visual neurons in the early visual system and has shown success in accurately predicting spiking responses in early sensory processing; however, most of the existing GLM-based modeling approaches rely on the assumption that the neural system is time-invariant. Although this may be a reasonable assumption for earlier sensory processing or functions, changes in the neurons’ spatiotemporal sensitivity which can occur in higher visual areas (Hegde, 2008) lead to nonstationary responses and computations that represent a time-varying stimulus–response relationship. The classical time-invariant GLM is thus inadequate for modeling the nonstationary characteristics of neurons in higher visual areas. Therefore, several studies have attempted to develop extensions of the GLM to incorporate changes in neuronal sensitivity over time, which have resulted in insightful hypotheses, experiments, or computational principles for investigating the neural basis of various behavioral and cognitive functions.

In this review, we provide an overview of the existing variations of GLMs, with a special focus on the time-varying extensions of GLMs. We discuss the advantages and limitations of these time-varying GLM-based methods and possible future directions in the context of characterizing the time-varying receptive fields of neurons in higher sensory areas and their role in generating flexible behavior. We also present other useful applications of GLM-based approaches, beyond providing an encoding model of responses. Specifically, we highlight the application of recent extensions to the GLM in describing both the encoding and decoding of time-varying information in higher visual areas, and in revealing possible neural mechanisms underlying our dynamic visual perception at the integration of sensory and motor signals. Finally, we discuss possible further development of GLM-based approaches and their connection to other modeling paradigms or modern machine learning methods.

## 2 GLM-based approaches for characterizing neuronal sensitivity

A sequence of neural spiking events occurring over time can be described as a binary point process time series, therefore, its statistical properties can be fully characterized by its conditional intensity function (CIF) (Brillinger, 1988; Daley and Vere-Jones, 2008). Given $H\left(t\right)$, which represents the spiking history of events up to time t and other related covariates, the CIF can be defined as


(1) $$\lambda \left(t|H\left(t\right)\right)=\lim_{\Delta \to 0}\frac{p\left\{N\left(t+\Delta \right)-N\left(t\right)=1|H\left(t\right)\right\}}{\Delta }$$

where $N\left(t\right)$ is the number of times the neuron fired a spike between time 0 and time t, with $t\in \left(0,T\right]$, and T representing the duration of the spike train. By binning the counting process, $N\left(t\right),$ over the entire time interval, we can construct a discrete-time representation of the point process, $r_{t}$, defined as


(2) $$r_{t}:=N\left(t\Delta \right)-N\left(\left(t-1\right)\Delta \right)$$

where Δ is the time bin size and $t\in \left\{1,2,\ldots ,\frac{T}{\Delta }\;\right\}$. Assuming the CIF is constant over any interval $\left(\left(t-1\right)\Delta ,t\Delta \right]$, and the time interval Δ is small enough such that there is at most one spike occurring within each interval, the probability of a spike happening in the time interval $\left(t,t+\Delta \right)$ can be approximated as


(3) $$p\left(\text{spike}\;\text{in}\;\left(t,t+\Delta \right)|H\left(t\right)\right)\approx \lambda \left(t|H\left(t\right)\right)\Delta$$

The sequence of discretized spiking response $r_{t}$ is typically modeled as a conditionally inhomogeneous Poisson point process, and the joint probability of the observed sequence is expressed as a product of Poisson probability mass functions as


(4) $$p\left(r|H\left(t\right)\right)=\prod_{t}p\left(r_{t}|H\left(t\right)\right)\propto \prod_{t}{\left(\lambda_{t}\Delta \right)}^{r_{t}}e^{-\lambda_{t}\Delta }$$

where $r:={\left\{r_{t}\right\}}_{t=1}^{\frac{t}{\Delta }}$, and $\lambda_{t}:=\lambda \left(t\Delta |H\left(t\Delta \right)\right)$.

Point process GLMs are a class of statistical models that represent the CIF, $\lambda_{t}$, as a nonlinear function applied to a linear combination of the covariates that may influence the spike generation (McCullagh and Nelder, 1989).

**Figure 1:**
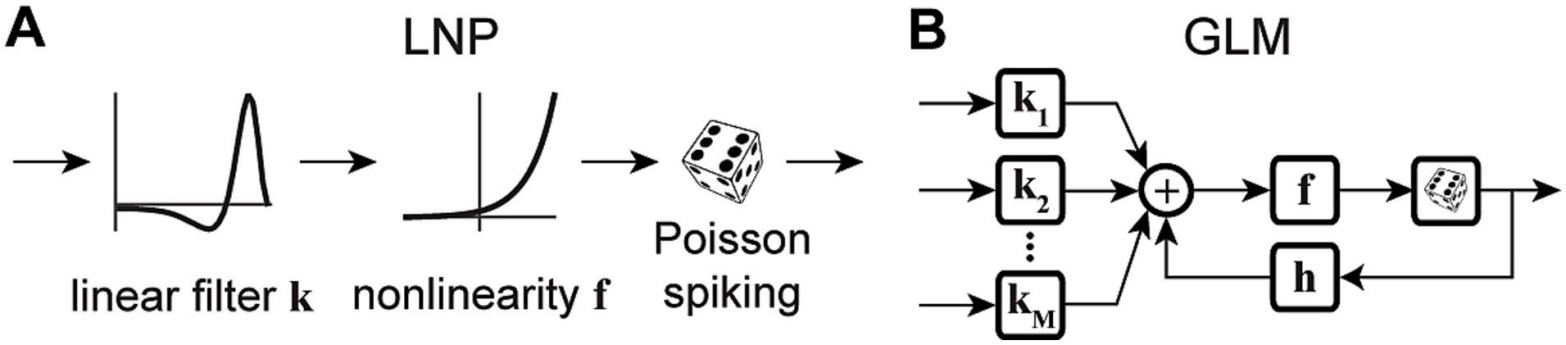
Schematic diagrams of an LNP model and a standard GLM. **(A)** Stimulus passes through a linear filter **k**, a static nonlinearity **f**, and a Poisson process generator to generate the spiking response. The model is strictly feedforward with no feedback term. **(B)** The GLM can receive multiple inputs comprising the intrinsic and extrinsic covariates related to the neuron’s response. Inputs pass through their corresponding linear filters **k**_**1**_, **k**_**2**_, …, **k**_**M**_, whose summed output passes through a nonlinearity **f**. The model incorporates a spike history term, through a post-spike filter **h**, that is fed back to the input of the link function **f**, which is typically chosen to be an exponential nonlinearity. The output of the nonlinearity representing the neuron’s instantaneous firing rate is used to produce spikes through a Poisson spike generator.

The simplest form of a GLM is a linear-nonlinear-Poisson (LNP) model (Figure 1A), which takes an input vector, usually representing a sequence of input stimuli, s, and projects it onto a vector of linear weights, k, known as the stimulus filter, representing the neuron’s receptive field (RF) (Paninski, 2003a; Rust et al., 2005; Perich and Miller, 2017). The filtered stimulus passes through a fixed nonlinearity, f, that outputs the instantaneous firing rate of the neuron, $\lambda_{t}$, which is used to produce the spike train via a Poisson process generator as


(5) $$\lambda_{t}\Delta =f\left(k^{T}s\right)$$

White noise stimuli are frequently used in reverse-correlation experiments for estimating an unbiased weight vector.

The LNP architecture provides a strictly feed-forward cascade description of sensory pathways. Although LNP models offer remarkable simplicity, studies have shown that spike history is crucial for accurate predictions of spiking activity, and has a considerable impact on the spiking dynamics (Pillow et al., 2005; Truccolo et al., 2005; Weber and Pillow, 2017). Moreover, there may be several extrinsic (stimuli, task variables, etc.) or intrinsic (spike history, concurrent ensemble activity, etc.) covariates that affect the spiking response of a neuron (Paninski, 2004; Simoncelli et al., 2004; Truccolo et al., 2005; Meyer et al., 2016). The CIF of a point process GLM can quantify the probability of spiking as a nonlinear function of multiple covariates, where the model parameters determine the form of the nonlinearity and the weights by which each covariate over different time lags contributes to the response generation. A common structure of a GLM framework is shown in Figure 1B, whose CIF can be specified as


(6) $$\lambda_{t}=f\left(\sum_{j}k_{j}^{T}x_{t}^{j}+h^{T}r_{t}^{\text{hist}}\right)$$

where $k_{j}$ denotes a linear weight vector that transforms the j^th^ covariate vector $x_{t}^{j}$ at different time lags with respect to time t. h denotes a post-spike filter capturing the spike history effects, and $r_{t}^{\text{hist}}$ denotes a spike history vector at time t. The first term of equation (6) implements a linear filtering process over individual model covariates. When the covariate x is chosen as a constant, its corresponding k incorporates a constant offset parameter, representing the baseline firing rate of the neuron. The net sum of all linear terms passes through a nonlinearity f, also referred to as the link function, to produce the neuron’s instantaneous firing rate $\lambda_{t}$, underlying the Poisson spiking process r. Note that this is also called a Poisson GLM; but GLMs can also use several non-Poisson distributions and link functions. It has been shown that the GLM parameters can be estimated efficiently in a maximum likelihood framework under some benign conditions on the nonlinearity function, and a single global optimum can be found using efficient gradient ascent methods (Paninski, 2004).

**Figure 2:**
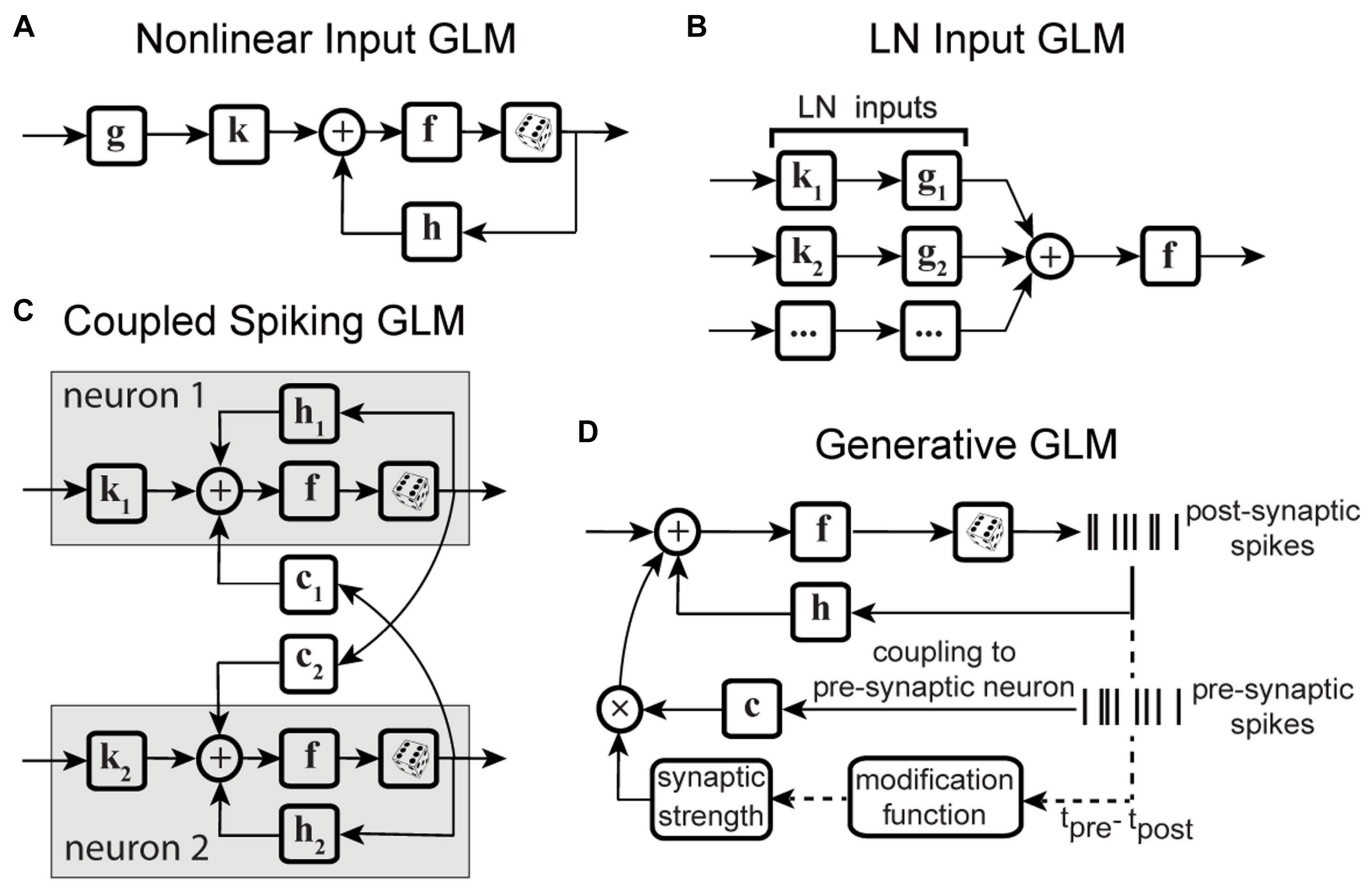
Schematic diagrams of example GLM variations. **(A)** Schematic of Nonlinear Input GLM. Stimulus passes through an input nonlinearity **g** which nonlinearly transforms the stimulus representation before applying linear filtering **k** and output nonlinearity **f** in a GLM structure (Ahrens et al., 2008) **h** represents the post-spike filter. **(B)** Schematic of LN Input GLM. Demonstrates a GLM with multiple linear filters (**k**_**1**_, **k**_**2**_, …) and nonlinearities (**g**_**1**_, **g**_**2**_, …) as LN inputs, also referred to as subunits, whose outputs are linearly combined and passed through a spiking nonlinearity *F*. These models can also be considered as two-stage LN models (McFarland et al., 2013). **(C)** Schematic of Coupled Spiking GLM. The GLMs for two neurons are linked to each other through interneuronal coupling filters **c**_**1**_ and **c**_**2**_ that capture each neuron’s response dependencies on spiking in the other neuron. **k**_**1**_ and **k**_**2**_ represent linear filters, and **h**_**1**_ and **h**_**2**_ represent post-spike filters for neuron 1 and neuron 2, respectively (Pillow et al., 2008). **(D)** Schematic of Generative GLM. The GLM of post-synaptic neuron is coupled to the pre-synaptic neuron through coupling filter **c** to an extent determined by the synaptic strength, which is modulated by the relative timing between pre- and post-synaptic spikes (**t**_**pre**_ **–** **t**_**post**_) through a spike-timing dependent modification function (Stevenson and Koerding, 2011).

Variations of point process GLMs have been widely used to study the functions of different cell types in the brain (Keat et al., 2001; Kim et al., 2011; Pozzorini et al., 2013; Park et al., 2014; Sheikhattar et al., 2018) (Table 1), and have shown the ability to replicate an extensive range of dynamic behaviors such as tonic spiking and bursting behaviors (Weber and Pillow, 2017). Linear-nonlinear (LN) models including a standard GLM assume that response nonlinearities can be captured after a linear filtering stage. However, there are processes throughout the neural system that can nonlinearly transform the inputs, including synaptic and dendritic nonlinearities or intermediate circuit nonlinearities that cannot be captured by a LN structure. Nonlinear input models can extend a GLM structure to incorporate a parametric nonlinear function of the input before the initial linear filtering stage (Figure 2A) (Ahrens et al., 2008; Willmore et al., 2016). To capture these input nonlinearities, the two-stage LN model or convolutional subunit model architectures have been introduced (Butts et al., 2007; Ahrens et al., 2008; Schinkel-Bielefeld et al., 2012; McFarland et al., 2013), where a weighted linear combination of several LN models passes through a final nonlinearity to generate responses (Figure 2B). GLMs can also be used to model multiple neurons through interneuronal coupling filters (Figure 2C), which provides important insights for understanding correlation structures within neuronal populations (Pillow et al., 2008). When incorporating interneuronal coupling filters, neural encoding demonstrates reduced noise, and the decoding process retrieves a greater amount of information. In addition to numerous applications to visual neurons, GLMs have also been adapted to study neurons in other brain regions such as auditory neurons (Calabrese et al., 2011; Sheikhattar et al., 2016) and somatosensory neurons (Pozzorini et al., 2013). One study used a GLM to drive synaptic plasticity in motor cortex by applying a coupling filter to the pre-synaptic neuron (Stevenson and Koerding, 2011), which was multiplied by a synaptic strength that changed over time (Figure 2D).

**Table 1:** Overview of the properties of the time-varying extensions of GLMs.

Model	Temporal modulation mechanism	Temporal resolution	Limitations related to time-varying properties	Example studies (neural systems and references)
Classical GLM	None (fixed linear filters)	Seconds when used in window-based time-varying models	Requires large time windows when used for time-varying models Limited to very slow dynamics	Retina, LGN (Keat et al., 2001) Sensorimotor cortex (Paninski, 2004) M1, dorsal premotor cortex (Stevenson et al., 2009; Perich et al., 2018) Retina (Gerwinn et al., 2010) LGN (Babadi et al., 2010) Auditory midbrain (Calabrese et al., 2011) M1 (Kim et al., 2011) Neocortex (Pozzorini et al., 2013) LIP (Park et al., 2014) A1 and prefrontal cortex (Sheikhattar et al., 2018) MT (Yates et al., 2020)
Nonlinear/LN input GLM	None (fixed linear filters)	Seconds when used in window-based time-varying models	Requires large time windows when used for time-varying models Limited to very slow dynamics	LGN (Butts et al., 2007) Barrel cortex (Ahrens et al., 2008) V1 (Kass et al., 2011) A1 (Ahrens et al., 2008; Schinkel-Bielefeld et al., 2012; Willmore et al., 2016) Retina, LGN, V1 (McFarland et al., 2013) Retina, V1 (Park et al., 2013)
Coupled spiking GLM	None (fixed linear filters)	Seconds when used in window-based time-varying models	Requires large time windows when used for time-varying models Limited to very slow dynamics	Retina (Pillow et al., 2008, 2011; Vidne et al., 2012) MT, LIP (Yates et al., 2017) Prefrontal cortex, mediodorsal thalamus (Rikhye et al., 2018) Thalamus, visual cortex, hippocampus, striatum, motor cortex (Zoltowski and Pillow, 2018) LIP, frontal eye field (Hart and Huk, 2020)
Population spiking GLM	None (fixed linear filters)	Seconds when used in window-based time-varying models	Requires large time windows when used for time-varying models Limited to very slow dynamics	M1 (Truccolo et al., 2005)
LFP-augmented GLM	LFP signals	Milliseconds with respect to LFP variation Time-invariant with respect to stimulus/input covariates	Limited to temporal dynamics of LFPs unless combined with other time-varying solutions	V1 (Kelly et al., 2010) A1 (Kayser et al., 2015)
Generative GLM	Short term plasticity modified coupling	Milliseconds with respect to spike dynamics Time-invariant with respect to stimulus/input covariates	Assumption of time-invariant filters Time variation limited to coupling term and spike timing plasticity	Motor cortex (Stevenson and Koerding, 2011)
Adaptive GLM	Adaptive filtering or adaptive priors	Deciseconds to seconds	Often requires regularization or large sample size	LGN (Stanley, 2002) Inferior colliculus, A1 (Meyer et al., 2014) A1 (Sheikhattar et al., 2016) Cortical assembly (Mukherjee and Babadi, 2021)
Nonstationary Gain GLM	Time-varying multiplicative gain	Milliseconds	Assumption of same gain for all response latencies	MT (Akbarian et al., 2018)
Sparse Variable GLM	Time-varying stimulus filters	Milliseconds	Sparsity measures needed for high-dimensional filters	MT (Niknam et al., 2017a,b, 2018, 2019) MT, V4 (Akbarian et al., 2021; Weng et al., 2023)
State space GLM	Hidden states	Milliseconds to seconds	Often requires population responses, large sample size, or the estimation of posterior densities	Hippocampus (Czanner et al., 2008) pIP10 neuron (Calhoun et al., 2019) Gustatory cortex (Escola et al., 2011) M1 (Shimazaki et al., 2012)

## 3 Benefits of GLM-based models for efficient representation of a high-dimensional stimulus space

One of the fundamental objectives in neuroscience is to characterize the neural code to understand how the brain represents and processes information. This has been challenging due to the high dimensionality of the sensory representation, which is also modulated by a number of non-sensory factors. To address this challenge, there are dimensionality reduction techniques, which represent a complex signal as a combination of a few key summarizing features that retain or emphasize specific aspects of interest within the data (Pillow and Simoncelli, 2006; Cunningham and Yu, 2014; Pang et al., 2016).

Moment-based methods for dimensionality reduction, such as the spike-triggered average (STA) or spike-triggered covariance (STC), can identify the directions in stimulus space along which the first or second-order statistics of the spike-triggered stimuli differ from those of all stimuli presented in the experiment, thereby identifying a lower dimensional stimulus subspace. The STA alone is sometimes not informative enough, and the STC is useful in these cases to find multiple relevant features (Rust et al., 2005). However, to provide an unbiased estimate of the stimulus dimensions, these methods rely on strong assumptions about the stimulus distribution. Other subspace methods such as principal component analysis (PCA) have been widely used in the analysis of neuronal population activity (Mazor and Laurent, 2005; Churchland et al., 2010; Ahrens et al., 2012). The simplicity of these strategies is appealing, nevertheless, their computational conditions such as independence between the stimulus dimensions make them limited for identifying a biologically relevant feature space representing the actual neural computations or pathways, or for capturing the complex nonlinear interactions between the identified dimensions (Paninski, 2003a,b; Pillow and Simoncelli, 2006; Fitzgerald et al., 2011).

To enable extracting nonlinear structures in the lower dimensional space, methods such as uniform manifold approximation and projection (UMAP) (McInnes et al., 2018) or *t*-distributed stochastic neighbor embedding (*t*-SNE) (Van Der Maaten et al., 2009) have achieved high performance in information decoding. Representational learning methods including those based on deep generative models (Yamins and DiCarlo, 2016), such as variational auto-encoders (VAEs) (Kingma and Welling, 2013; Pandarinath et al., 2018), provide another set of nonlinear tools for dimensionality reduction at the level of neural population dynamics. These methods show promise for extracting complex nonlinear structures in the neuronal population, nevertheless, the lower dimensional latent spaces extracted using these models are often difficult to interpret and so fall short in identifying the underlying neural mechanisms (Humphries, 2021).

Alternatively, information-theoretic methods based on mutual information or entropy have been used to determine the dimensions along which stimulus information reflected in the response is maximized (Paninski, 2003a; Sharpee et al., 2004; Shlens et al., 2006; Tang et al., 2008). The maximum mutual information methods can analyze neurons in higher-level sensory cortex that respond to non-Gaussian naturalistic stimuli instead of white noise, but demand a large amount of data for accurate estimation (Sharpee et al., 2004). However, these methods are once again challenged by the dimensionality problem, where the number of parameters required to specify the density functions grows exponentially with the number of stimulus dimensions. Combining both the STA/STC and mutual information methods has proven to be effective for dimensionality reduction (Pillow and Simoncelli, 2006). This technique maximizes information relying exclusively on the first and second moments of the spike-triggered stimulus ensemble to improve tractability, but may miss information in the higher order moments.

In the context of LN cascade models, likelihood-based approaches provide a powerful framework to robustly estimate multiple stimulus dimensions and characterize their nonlinear relationship and can also scale well with the dimensionality of the stimulus. Parameterizing the linear stimulus filters and the nonlinearity using a set of basis functions can transform the stimulus–response relationship to a much lower dimensional space. The linear filters, referred to as kernels of the neuron, are then expressed as a weighted sum of the basis functions. In this context, extending the GLM framework to incorporate nonlinear covariate terms over a projection of the stimulus onto multiple linear filters enables capturing of multi-dimensional stimulus–response relationships (Ahrens et al., 2008; Park et al., 2013).

A high-dimensional representation of the neurons’ spatiotemporal kernels is necessary to capture the rapid and intricate changes in the spatiotemporal sensitivity of neurons in higher visual areas. The key lies in identifying the lower dimensional subspace that describes the dynamic input–output relationship with high temporal precision, in an assumption-free and data-driven manner. Existing approaches to address this challenge are discussed in the next section, with a focus on time-varying multi-filter extensions of GLMs.

## 4 GLM-based approaches for nonstationary responses

It is computationally challenging to model neurons in the higher visual areas where various behavioral, motor, or cognitive modulatory factors create fast and nonlinear dynamics in the neuron’s spatiotemporal sensitivity, which results in a high-dimensional stimulus representation. These time-varying modulations and nonlinear spatiotemporal dynamics lead to a complicated nonstationary stimulus–response relationship. Consequently, many conventional computational approaches that rely on assumptions of time invariance and linear processing of stimuli within neurons’ classical RF prove inadequate to capture activity in higher visual areas. Reverse-correlation techniques based on analyzing the cross-correlation between the stimulus and the recorded neural responses to white noise stimuli, have been successful in describing the neuron’s input–output relationship. Particularly, the application of reverse-correlation techniques on the neuronal data collected from early visual areas during spatiotemporal white noise stimulus presentation, has improved our understanding about the functions of neurons and their receptive field properties in early stages of visual processing. However, these methods assume that the neuron’s function remains invariant over time which results in a static model of their spatiotemporal RF properties (Stanley, 2002). Although the time-invariance assumption for the spatiotemporal RF properties in the early visual pathway may be valid for several visual functions, it fails to capture the time-varying dynamics in the neural responses throughout the visual pathway which underly various dynamic behaviors (Fuster and Alexander, 1971; Gottlieb et al., 1998; Bisley and Goldberg, 2003; Ikeda and Hikosaka, 2003; Womelsdorf et al., 2006; Mitchell et al., 2007; Herrero et al., 2008; Anton-Erxleben et al., 2009; Moore and Chang, 2009; Kok et al., 2012; Mante et al., 2013; Saleem et al., 2013; Noudoost et al., 2017). Recent work has acknowledged this issue and developed new approaches to characterize time-varying neural responses on the timescale of tasks and behaviors.

One simple approach is dividing the overall nonstationary responses into discrete time intervals to be modeled separately (Weinberger, 1993; Mehta et al., 1997; Gandolfo et al., 2000; Fritz et al., 2003; Sharpee et al., 2006; Meyer et al., 2014). This approach assumes that the stimulus–response relationship is fixed during each time interval (or at least settles for describing the average stimulus–response relationship in that window). A study of hippocampal place fields in rats quantified the place field size separately on each lap of running, and showed field size increasing over time (Mehta et al., 1997). Another study investigated how monkeys adapt to a new environment by dividing the trials into epochs and observing the change of activity over epochs (Gandolfo et al., 2000). These studies track changes in the RF on the order of minutes, while some achieve higher resolution (5-20 s) by assuming small fluctuations in RF parameters over consecutive segments (Meyer et al., 2014). Splitting data into segments provides estimates of the neural states in specific time windows, but cannot analyze activity continuously on shorter timescales. Similarly, studies investigating saccadic modulations of visual responses have often compared neural sensitivity between two fixation and perisaccadic periods, or across discrete time windows of fixed length relative to the onset or offset of a saccade (Bremmer et al., 2009; Zanos et al., 2016). As the desired time resolution with this approach increases, the amount of data required to estimate neural sensitivity also increases. Moreover, independent estimation of neuronal sensitivity over discrete windows cannot trace the continuous RF evolution over time at the same temporal resolution as the saccade-induced visual response modulations.

**Figure 3:**
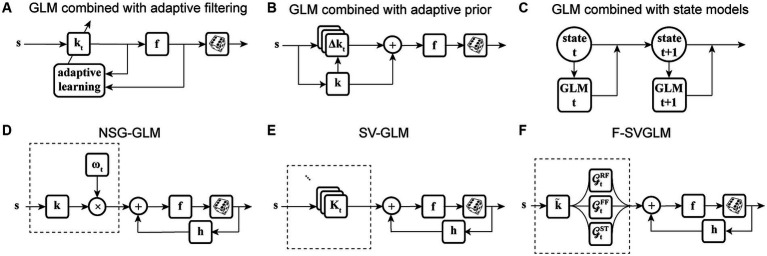
Time-varying extensions of GLM. **(A)** GLM combined with adaptive filtering. Stimulus **s** passes through an adaptive filter **k**_**t**_ and nonlinearity **f** to generate spikes through a stochastic process. The filter **k**_**t**_ is estimated by adaptive learning algorithms such as recursive least-squares or recursive maximum likelihood estimation with possible regularization, for example, to impose sparsity on the filter parameters. The nonlinearity **f** and the spike process generator can use multiple link functions or distributions. **(B)** GLM combined with adaptive prior. Stimulus **s** passes through a linear filter which is a linear combination of a fixed filter **k**, representing the long-term static RF of the neuron, and a temporally localized deviation term **Δk**_**t**_, representing the difference between **k** and time-dependent estimate of local RF **k**_**t**_. The static filter is used as a prior for maximum *a posteriori* estimation of local filters over fixed discrete time windows. The net filtered output passes through nonlinearity **f** and spiking process. **(C)** GLM combined with state models. At each timepoint **t**, a discrete hidden state is specified by a set of GLMs that predict the state’s output and the transition probability. Each hidden state (**state t**, **state t + 1**) has a corresponding set of GLMs (**GLM t**, **GLM t + 1**). **(D)** Nonstationary Gain GLM (NSG-GLM). The output of linear filtering, using a parametric linear filter **k**, is multiplied by a time-varying gain factor $\omega_{t}$. The resulting signal, representing the neuron’s time-varying sensitivity, is summed with a spike history term through post-spike filter **h**, and passed through nonlinearity **f** and Poisson spiking. **(E)** Sparse Variable GLM (SV-GLM). Estimates time-varying spatiotemporal kernels **K**_**t**_ which represent four-dimensional parametric kernels, across space, delay, and time dimensions, whose parameters are jointly fitted over the duration of individual trials. These four-dimensional kernels **K**_**t**_ capture the time-varying spatiotemporal sensitivity of the neuron on the timescale of a few milliseconds. The post-spike filter **h**, nonlinearity **f**, and spike generator blocks are similar to those used in a standard GLM. **(F)** Factorized Sparse variable GLM (F-SVGLM). Kernels **K**_**t**_ estimated from the SV-GLM (E) can be factorized as a mixture of multiple time-varying spatiotemporal gaussian kernels whose parameters are estimated in a least-squares sense. This factorization can, for example, identify and characterize three sources of spatiotemporal sensitivity modulations during saccades associated with RF, FF, and ST areas $G_{t}^{RF},$ $G_{t}^{FF},$ and $G_{t}^{ST}$ which modulate the static spatiotemporal kernel $\tilde{k}$ over time to saccade with high temporal resolution.

The second category of nonstationary models considers the continuous temporal evolution of the underlying system. One of the most commonly used solutions is recursive methods for filter estimation, which allows for temporal variations of the model parameters (Frank et al., 2002; Stanley, 2002; Eden et al., 2004; Ergün et al., 2007; Sheikhattar et al., 2016). Extending the GLM structure to integrate these recursive approaches, such as recursive least-squares filtering (Stanley, 2002) or maximum likelihood adaptive filtering (Sheikhattar et al., 2016) (Figure 3A), or using adaptive priors for maximum *a posteriori* estimation of temporally localized filters (Meyer et al., 2014) (Figure 3B), has generated powerful models capable of capturing the temporal dynamics of neurons’ RFs in several neural systems. For example, the adaptive approach was used to track the spatiotemporal changes of RFs in the early visual system using time-varying weighting functions (Stanley, 2002). A similar approach was also applied to characterize the spectrotemporal RF plasticity in the primary auditory cortex (A1) with high temporal resolution (Sheikhattar et al., 2016). While these approaches can account for time-varying sensitivity of neurons, the amount of data required for robust estimation of these adaptive models could vary substantially depending on the size of their parameter space. To increase the robustness of estimating a high-dimensional parameter space, the existing adaptive filtering approaches often use standard regularization techniques such as lasso regression (L_1_-norm) (Tibshirani, 1996; Sheikhattar et al., 2016) and ridge regression (L_2_-norm) (Hoerl and Kennard, 1970; Meyer et al., 2014). However, these regularization techniques for dimensionality reduction do not use behavior to guide the learning of significant dimensions or model parameters, which may not provide the dimensions contributing to response generation underlying certain time-varying behavior.

State-space models are another class of models frequently applied to analyze neural systems with nonstationary characteristics. In state-space models, the subsequent state of the system is established by considering both its present state and the input it receives. Some studies have used linear dynamical systems (Czanner et al., 2008; Cunningham et al., 2009; Shenoy et al., 2013), while others adapted the traditional hidden Markov models (Paninski et al., 2010; Escola et al., 2011; Shimazaki et al., 2012; Kao et al., 2017). There are state-space models based on extending a GLM structure to include hidden state variables, for example, by combining a GLM and a hidden Markov model (GLM-HMM) (Escola et al., 2011), which determine the temporal evolution of the neuron’s stimulus–response function, using state-dependent stimulus filters. Modifying the GLM-HMM to use categorical outputs, Calhoun and colleagues applied these models on behavioral data to predict the discrete behaviors performed by an animal. This state-dependent GLM allowed for time-varying probabilities of transitioning and actions to incorporate the effect of internal states of the system on sensorimotor processing over time (Figure 3C) (Calhoun et al., 2019). Although these studies succeeded in modeling time-varying systems and decoding neural dynamics, they often rely on population responses or estimating the posterior densities, which make them insufficient for capturing time-varying stimulus sensitivity at the level of single neurons or on fast timescales where the data is limited.

Each approach mentioned above has its own pros and cons, but few of them can succeed in capturing the responses of neurons in the higher visual areas where multiplexed neural signals reflecting the interaction of sensory and non-sensory variables complicate the neural code (Table 1). One such example is saccadic eye movements, which involve fast and complex modulations in neuronal sensitivity in the extrastriate visual areas and prefrontal cortex during the perisaccadic period (Nakamura and Colby, 2002; Sommer and Wurtz, 2006; Zirnsak et al., 2011, 2014; Neupane et al., 2016, 2020; Niknam et al., 2019; Akbarian et al., 2021). Quantitatively characterizing the perisaccadic sensitivity modulations on the fast timescale of saccades is critical for understanding the neural basis of various perisaccadic perceptual phenomena. Studies have shown that neurons in the MT and V4 areas experience a reduction in sensitivity to stimuli that appear within their RFs shortly before a saccade, a phenomenon known as saccadic suppression. Neurons in extrastriate and prefrontal areas have also been observed to preemptively shift their RF to the postsaccadic RF (future field remapping), or to the saccade target (saccade target remapping) prior to saccade onset.

Motivated by the findings on perisaccadic response modulations and the limitations of the existing methods to characterize and trace those fast and nonlinear dynamics, a recent study developed a series of nonstationary extensions of a GLM to capture time-varying visual sensitivity. The first extension, referred to as Nonstationary Gain GLM (NSG-GLM), uses multiplicative temporal gain kernels to capture the neuron’s sensitivity to each spatiotemporal feature as varying across a saccade and devises a robust alternating maximum likelihood optimization procedure under a Poisson probability distribution (Figure 3D) (Akbarian et al., 2018). Although the NSG-GLM can account for perisaccadic modulatory gain effects, it is unable to reproduce the perisaccadic response changes that require more than a simple instantaneous gain mechanism, such as alterations in response latency. The subsequently developed Sparse Variable GLM (SV-GLM) is able to capture a wider variety of modulations by implementing time-varying stimulus kernels representing the neuron’s perisaccadic spatiotemporal RF dynamics (Figure 3E) (Niknam et al., 2019). The SV-GLM can be viewed as a set of GLMs that are simultaneously fitted, where each GLM corresponds to an individual time point, which allows it to represent the temporal evolution of the system on a millisecond-timescale with no assumptions about the functional form of the temporal modulations. Moreover, the SV-GLM estimation implements a dimensionality reduction procedure that identifies a sparse representation of high-dimensional perisaccadic stimulus kernels by directly quantifying the statistical significance of individual RF parameters, which enables robust parameter estimation despite the limited data available during the perisaccadic period. This assumption-free approach offers accurate estimations of perisaccadic modulations, and identifies specific times and locations where neural sensitivity changes, allowing potential interpretations of the response modulations at a mechanistic level. Such a comprehensive and unbiased description of the RF dynamics enables the identification of potential sources responsible for the spatiotemporal sensitivities reflected in the kernels of the SV-GLM. A parsimoniously factorized version of the SV-GLM (F-SVGLM) is then developed to capture the perisaccadic modulations originating from either the RF, FF, or ST sources via a mixture decomposition procedure optimized in a least squares sense (Figure 3F) (Niknam et al., 2019).

**Figure 4:**
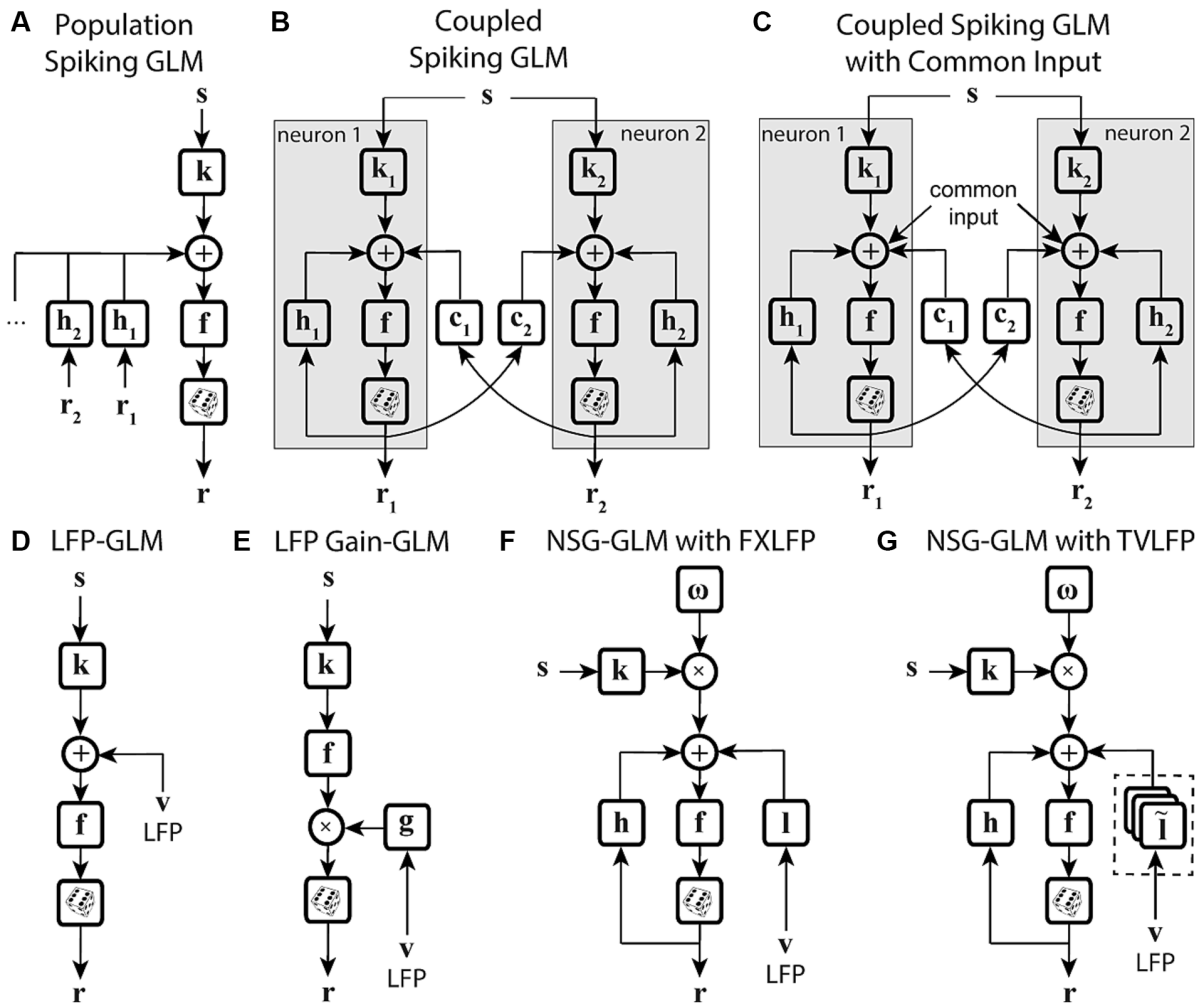
State-dependent extensions of GLMs using population-level activity. **(A)** Spiking GLM incorporating covariates related to neuronal ensemble spiking activity. Stimulus **s** passes through linear filter **k**, the filtered signal is summed with the outputs of post-spike filters **h**_**1**_, **h**_**2**_, etc., which capture the effects of population spike histories from neuron 1’s response **r**_**1**_, neuron 2’s response **r**_**2**_, etc. recorded simultaneously. The neuron’s own spiking history **r** can also be incorporated using an additional post-spike filter (not shown). The resulting combined signal passes through nonlinearity **f** and spike generator to produce response **r**. **(B)** Spiking GLM incorporating covariates related to other neurons’ coupled spiking activity. Similar to Figure 2C. **k**_**1**_ and **k**_**2**_ represent linear filters, **h**_**1**_ and **h**_**2**_ represent post-spike filters, **c**_**1**_ and **c**_**2**_ represent coupling filters, and **r**_**1**_ and **r**_**2**_ represent responses for neuron 1 and neuron 2, respectively. Interneuronal coupling filters capture correlated activity of simultaneously recorded neurons; **c**_**1**_ receives input from neuron 2, while coupling filter **c**_**2**_ receives input from neuron 1. **(C)** Spiking GLM incorporating a hidden state-dependent common input covariate. Similar to (B), but with common input added to the output of the linear filters **k**_**1**_ and **k**_**2**_, along with the output of the coupling filters **c**_**1**_ and **c**_**2**_, and the output of the post-spike filters **h**_**1**_ and **h**_**2**_. The common input variable reflects the effects of common noise shared between the simultaneously recorded neurons. **(D)** Spiking GLM incorporating LFP activity. Stimulus **s** passes through linear filter **k**, whose output is summed with simultaneously recorded LFP values **v**, and the net signal is used to generate spiking response **r** through nonlinearity **f** and spike generator. LFP activity represents an aggregate signal reflecting the state of the network. **(E)** Spiking GLM incorporating LFP-controlled gain modulation. Stimulus **s** passes through linear filter **k** and nonlinearity **f**. The resulting firing rate is multiplied by a gain factor **g**, which is controlled by the phase or power of LFP **v**, to produce response **r**. A discrete set of LFP phase and power values are used to specify multiple oscillatory states of the network. These states can also control a background firing term through an additive state-dependent offset variable (not shown). **(F)** Time-varying spiking GLM incorporating LFP as an internal covariate. Extended form of the NSG-GLM structure in Figure 3D, where information from LFP **v** is incorporated through a time-invariant kernel **l** for spike rate prediction through nonlinearity **f**. FXLFP denotes a fixed LFP filter over the course of a trial, which captures how network-level activity modulates individual neuron’s spiking. **(G)** Time-varying spiking GLM incorporating LFP as an internal nonstationary covariate. Similar to **(F)**, but LFP information **v** is modeled through a time-variant temporal kernel $\tilde{l}$. TVLFP denotes a time-varying LFP filter, which captures the dynamics by which network-level activity can variably modulate an individual neuron’s spiking over the course of a trial.

Another potential way to improve a model’s ability to capture time-varying sensitivity is to incorporate signals related to the overall state of the system, in the form of population activity (Table 1). Truccolo and colleagues have constructed a point process GLM structure which takes concurrent ensemble spiking activity in primary motor cortex (M1) as one of its input covariates (Figure 4A) and have shown its improved prediction and decoding performance over standard GLM models (Truccolo et al., 2005). The GLM architecture that integrates interneuronal coupling filters (Figure 4B) and one that additionally combines common input latent variables (Figure 4C) are also examples of GLM extensions capable of assimilating population-level information when estimating the stimulus kernels (Pillow et al., 2008; Vidne et al., 2012). These extensions have taken advantage of the simultaneous recording of multiple neurons using multielectrode arrays. Population-level information can also be reflected in aggregate brain signals such as local field potentials (LFPs). There are models extending the GLM structure to capture different aspects of LFP signals, such as including a vector of LFP values as an input term (Figure 4D) (Kelly et al., 2010), or incorporating state-dependent gain and offset variables determined by the LFP’s phase or power (Figure 4E) (Kayser et al., 2015). Although the output of these models depends on the population state changes, their stimulus sensitivity parameters are time-invariant and thus are not able to characterize the time-varying characteristics of the neuron’s RF. Building upon the development for time-varying GLM extensions, Niknam and colleagues extended the spiking NSG-GLM (Figure 3D) to incorporate the simultaneously recorded LFP as a model covariate with its own temporal filter (Figures 4F,G) (Niknam et al., 2018). This approach enabled the characterization of the time-varying spatiotemporal sensitivity of extrastriate neurons during saccades and demonstrated increased prediction and decoding performance compared to the previously developed NSG-GLM (Niknam et al., 2017a,b, 2018).

## 5 Decoding time-varying sensory information using GLM frameworks

Understanding how brain activity generates our behavior requires deciphering the algorithms by which the neural representations in sensory areas shape perception or cognition. Decoding methods seek to infer input stimuli from neural responses. Existing statistical decoders are primarily based on statistically optimal inference methods (Seung and Sompolinsky, 1993; Salinas and Abbott, 1994; Britten et al., 1996; Warland et al., 1997; Ma et al., 2006), which can provide an upper bound for performance, and generally require a full knowledge of task variables and response and noise properties, and may not be assumed to represent biologically-plausible decoding strategies.

An alternative decoding approach models neural dynamics using state-space methods to decode behavioral or motor variables that can vary over time (Shenoy et al., 2013). Typical state-space models assume that behavioral measurements such as movement kinematics, or decision choices at each point in time are directly represented in the neural activity at that time (Churchland et al., 2012; Kao et al., 2014; Gallego et al., 2018; Wallis, 2018). Another approach for state-space decoders models neural dynamics in terms of a latent variable that constitutes the state in the model (Kemere et al., 2008; Wu et al., 2009; Cunningham and Yu, 2014; Golub et al., 2015; Aghagolzadeh and Truccolo, 2016; Sani et al., 2018, 2021). Many state-space decoders, such as a Kalman filter, take as input the spike counts in relatively large time bins and are often based on population responses. These decoders have been very successful in neural prostheses or brain machine interface systems (Shenoy, Sahani and Churchland, 2013; Nuyujukian et al., 2017; Wallis, 2018; Shanechi, 2019; Willett et al., 2021). A more recent decoding framework that also leverages the latent variable approach employs a neural network encoder, CEBRA, which uses both behavioral and neural data to make inferences about the latent variables for decoding (Schneider et al., 2023).

Statistical models provide an alternative framework for inferring about stimulus variables using inverse models. Point process regression-based models such as GLMs, particularly those with generalizable capability to predict responses to unseen input patterns, allow the derivation of optimal estimators for reconstructing input stimuli from single trial spike trains. These models describe spiking responses of a neuron **r** to a given stimulus **s** through the conditional probability *p* (**r**|**s**), and can then be inverted to provide an optimal readout of sensory information from single-trial spike trains, for example, by estimating the posterior probability *p* (**s**|**r**) via Bayes’ rule (Paninski et al., 2007). The variations of GLMs mentioned in previous sections prove the power of this statistical modeling framework in capturing high-dimensional and nonlinear relationships between neural responses and multiple internal and external covariates on the same timescale as tasks or behaviors. These time-varying models allow for developing model-based decoders that can dynamically read out sensory or behavioral information from time-varying response characteristics in higher cortical areas (Park et al., 2014; Aoi et al., 2020; Akbarian et al., 2021; Weng et al., 2023).

Encoding and decoding approaches are frequently used in concert; for example, an encoding model is established based on physiological recordings of neural responses, and a model-based decoding method can then be employed to link neuronal responses to the readout of input stimuli (Pillow et al., 2008, 2011; Valente et al., 2021).

## 6 Mechanistic-level interpretation of neuronal activity using combined GLM-based encoding and decoding approaches

**Figure 5:**
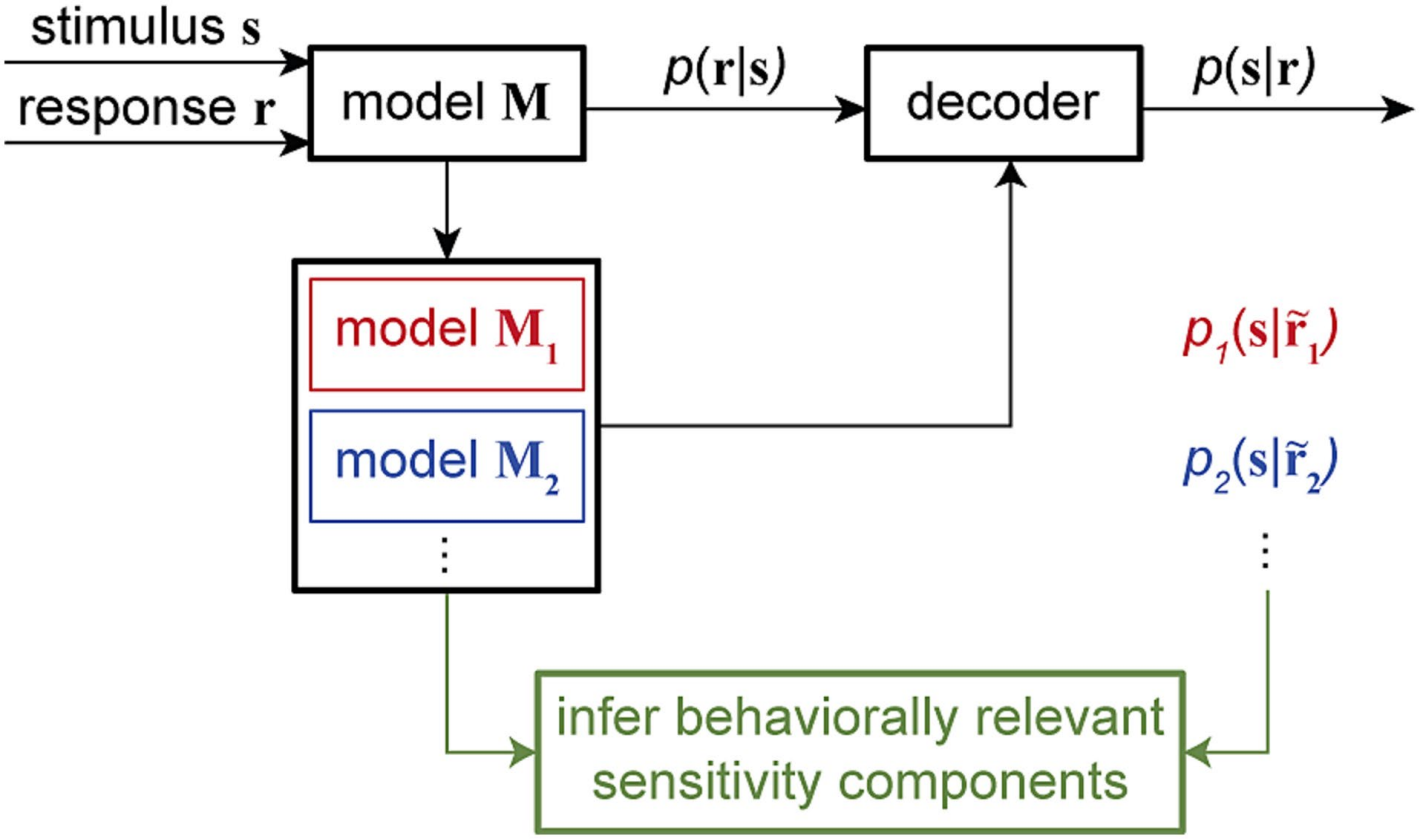
Combined encoding and decoding framework for mechanistic-level understanding of perception. The encoding model **M**, fitted using the input stimulus **s** and the output response **r** to assign the conditional probability *p* (**r**|**s**), can be used to develop a decoder for reading out the stimulus from a given response, for example, by evaluating the stimulus posterior distribution *p* (**s**|**r**). Through a systematic model manipulation procedure, an encoding model **M** can be transformed into different altered models (model **M**_**1**_, model **M**_**2**_, …), generating altered responses ${\tilde{r}}_{1},$ ${\tilde{r}}_{2},$ … Each altered model can create a readout of the stimulus *p_1_*(**s**|${\tilde{r}}_{1}),$ *p_2_*(**s**|${\tilde{r}}_{2}),$… through the decoder. By corresponding alterations in the readout to alterations in the model, this approach can provide an unsupervised procedure to screen for the neuron’s sensitivity components underlying certain readout outcomes. This combined encoding and decoding framework can therefore link the neuronal representation of sensory information to perception or behavior.

By manipulating model components in ways that are impossible in physiological experiments, these combined encoding-decoding approaches offer a powerful means to investigate specific hypotheses about the possible mechanisms through which downstream neurons can read out the encoded information. Particularly, by quantifying the time-varying dependency of single-trial spiking responses on multiple components of sensitivity modulations and providing a dynamic readout of various types of information, time-varying GLM frameworks can be used to examine the neural basis of various behaviors across timescales. Computationally manipulating the neuron’s sensitivity components and analyzing the associated model-predicted readouts can disentangle the contribution of various response components underlying specific perceptual or cognitive phenomenon (Figure 5).

**Figure 6:**
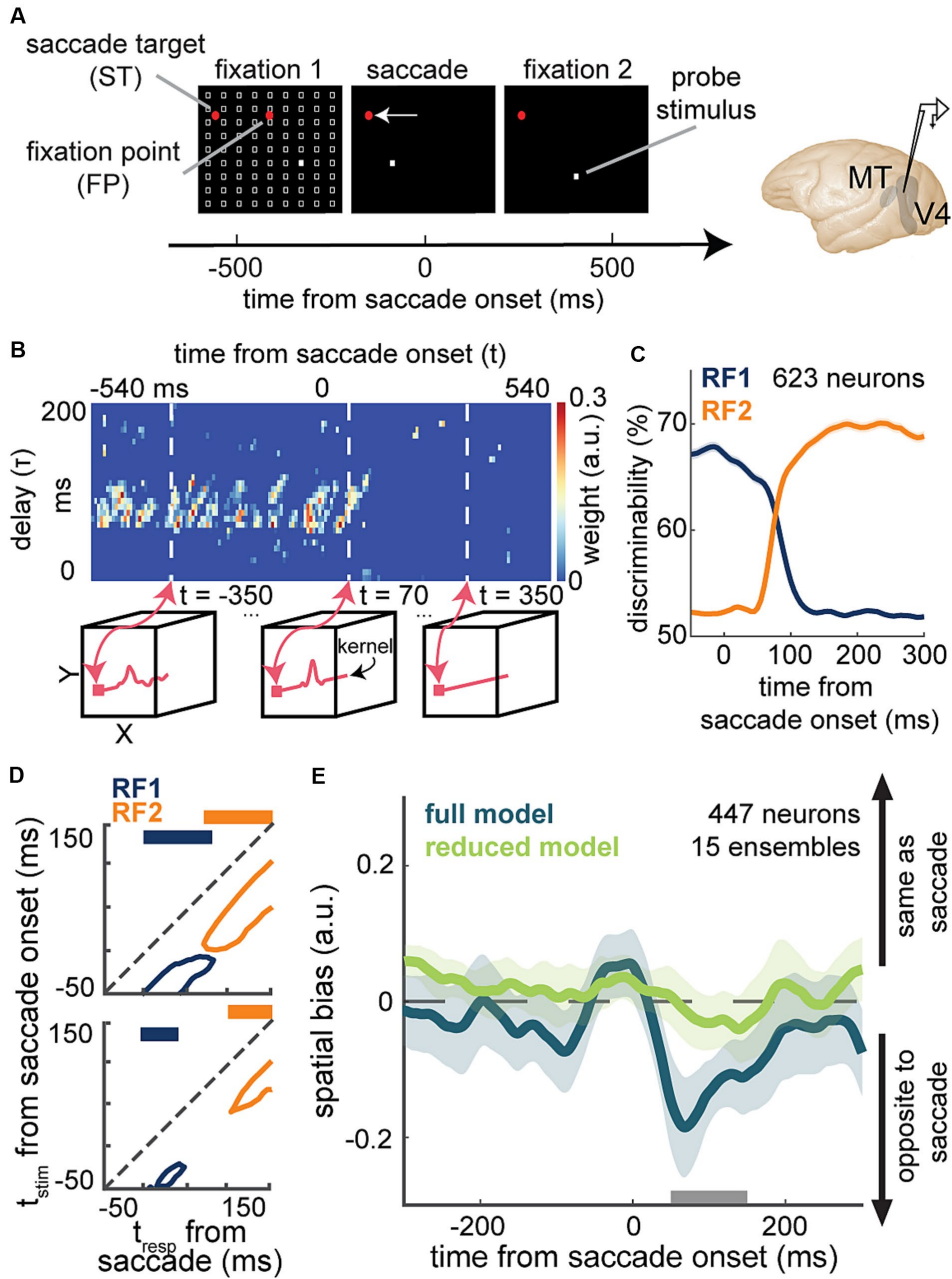
Application of a time-varying GLM framework for encoding and decoding of visual information during saccadic eye movements. **(A)** Schematic of the visually-guided saccade task with probes. After monkeys fixate on a central fixation point (FP), a saccade target (ST) point appears (horizontally displaced from the FP in either direction). After a randomized time from 700 to 1,100 ms, the FP disappears and the animal saccades to the ST and maintains fixation there. Throughout the first fixation, saccade, and second fixation, a series of 7-ms probes appear in a pseudorandom order in a 9 × 9 grid of possible locations (white squares). Neurons were recorded from MT or V4. **(B)** Shows weight values for a stimulus kernel, across time and delay, at the RF1 center probe for a sample neuron, estimated through an SV-GLM fit on the recorded spiking data. Each kernel captures the neuron’s sensitivity in spatial (x, y), time (t, time of response relative to saccade onset), and delay (τ, time of stimulus relative to response) dimensions. Line plots in the bottom boxes show the cross-sections of the above map at the specified times. **(C)** The temporal dynamics of neurons’ spatial discriminability across time from saccade onset around RF1 (blue) and RF2 (orange) locations, obtained using a decoder, trained on the model-predicted responses to a pair of probe locations around RF1 and RF2 [related to Figure 3 in Weng et al. (2022)]. **(D)** Discriminability for probes in RF1 (blue) and RF2 (orange) before (top) and after manipulating the model through nulling integration-relevant kernel components (bottom). Replacing the weights of these integration-relevant kernel components with the fixation weights led to a gap in sensitivity. The projections on top show whether there is a gap in sensitivity [related to Figure 2 in Akbarian et al. (2021)]. **(E)** Mean spatial bias in reading out the location of a perisaccadic stimulus around the ST area, obtained using the similarity of population kernel vectors corresponding to a center probe location versus its 8 neighboring probes across 15 neuronal population ensembles over time from saccade onset. The spatial bias captured by the full model (dark green) was eliminated in the reduced model with bias-relevant kernel components removed (light green). Spatial bias is significantly smaller in the reduced model in the period indicated by the gray bar (50-150 ms after saccade onset) related to Figure 3 in Weng et al. (2023).

A recent study by Akbarian and colleagues is one of these examples in which an encoding model capturing the change of neuronal sensitivity across saccades with high temporal resolution allowed a model-based decoder that linked perisaccadic modulations in neuronal sensitivity to saccade-induced changes in the perceived visual stimuli at each instant of time (Akbarian et al., 2021). Visually-guided saccade tasks are commonly used to study changes in perception during saccades (Kaiser and Lappe, 2004; Michels and Lappe, 2004; Hamker et al., 2008; Binda et al., 2009; Rolfs and Carrasco, 2012). The subject fixates on a fixation point and a second visual point called the saccade target appears. When the fixation point disappears, the subject makes a saccade to the saccade target. The SV-GLM framework (Figure 3E), fitted on spiking responses from visual areas during a visually-guided saccade task with pseudorandom visual stimulation (Figure 6A), provided an unbiased model capturing the time-varying spatiotemporal sensitivity of MT and V4 neurons during saccades (Figure 6B).

The model’s ability to predict spiking responses to arbitrary input stimuli, enabled a model-based decoder to read out stimulus information at a particular location, latency, and time relative to saccade onset. The model-predicted responses to probe stimuli presented around the presaccadic RF (RF1) and postsaccadic RF (RF2) were used to measure location discriminability at each time point relative to saccade onset and for each stimulus delay relative to that time point [Figure 6C; related to Figure 3 in Weng et al. (2022)]. This study was one of the first to demonstrate the capability of a GLM-based time-varying model in decoding visual stimuli from the instantaneous neuronal sensitivity in higher visual areas, operating on the fast timescale of a visual behavior. In line with the subjective perception of a seamless visual scene, the decoder revealed that there is no point in time during saccades when spatial sensitivity is entirely lost (Figure 6D top).

To pinpoint the basis of a continuous visual representation across saccades, Akbarian and colleagues devised an unsupervised procedure to screen for integration-relevant sensitivity components based on a model manipulation that led to a gap of sensitivity, a period when the neuron could not discriminate probes at either RF1 or RF2 (Figure 6D bottom). This combined encoding and decoding approach could identify the response components necessary for an uninterrupted stimulus readout across saccades. This model-based approach was thus able to link perisaccadic responses to perception, revealing a potential neural basis for transsaccadic spatial integration. Using a similar approach based on the manipulation of the fitted SV-GLM kernels, Weng and colleagues were able to identify the essential extrastriate sensitivity components responsible for driving a spatial bias in the localization of perisaccadic stimuli (Figure 6E), which represents another example of using model-based computational manipulations to link neural responses to perception (Weng et al., 2023).

Moreover, there are additional instances showcasing the utilization of combined GLM-based encoding and decoding for generating a mechanistic-level comprehension for various behaviors. Park and colleagues investigated neural encoding in the lateral intraparietal area (LIP) by analyzing spike trains with a GLM, effectively predicting spike trains for individual trials of a decision-making task. Furthermore, they developed an optimal decoder suitable for implementation in biologically plausible circuits and discovered that optimal decoding requires the integration of LIP spikes over two distinct timescales (Park et al., 2014). Yates and colleagues trained linear decoders on small ensembles of simultaneously recorded MT neurons to distinguish motion direction with and without awareness of interneuronal correlations. Additionally, they used a GLM-based analysis on the decoder’s choice output to evaluate the impact of each pulse on the overall decoder output. Their findings illustrate that the simple decoder, without factoring in correlations, replicates psychophysical performance and achieves optimal sensitivity (Yates et al., 2020).

## 7 Applications of GLM-based approaches beyond encoding and decoding models

The ability of the GLM to capture the statistical dependency of neural responses to multiple intrinsic and extrinsic covariates at the level of single trials and individual neurons provides a powerful platform to infer circuit-level computations and network-level properties. Neural computations underlying our behavior rely on the coordinated activity of neuronal ensembles and their communications. On the other hand, recent advances in large-scale optical or electrophysiological recording such as the use of Neuropixels have generated valuable datasets capturing the simultaneous activity of many neurons within individual or across multiple regions in the brain (Jun et al., 2017).

Interpreting these datasets will require developing tools capable of capturing the complex connectivity and communication patterns in neuronal ensembles in a statistically efficient, functionally interpretable, and computationally tractable manner. Although quantitative measures based on pairwise correlations or distances of spike trains have generated a greater understanding of the properties of coordinated activities, interareal communications, and their possible functional implications, those approaches face key challenges for capturing higher order interactions or requiring a large number of trials for robust calculation of those measures. Statistical model-based approaches can address these challenges by capturing the statistical dependencies between responses (Truccolo et al., 2010; Kass et al., 2011; Roudi et al., 2015; Mukherjee and Babadi, 2021). A GLM-based framework is a strong candidate for inferring circuit-level computations from the models of individual neurons and their interactions with no prior mechanistic level assumptions (Pillow et al., 2008; Truccolo, 2016; Zoltowski and Pillow, 2018). For example, by analyzing the amplitude, sign, shape, or time-lags of interneuronal coupling filters, one can characterize the strength, excitatory versus inhibitory, temporal dynamics, or directed dependency properties of the connections between neurons (Keeley et al., 2020). The application of GLMs can be expanded to analyze neural activity from several simultaneously recorded areas through regression-based methods or shared latent variable approaches (Keeley et al., 2020). In regression-based models, interactions are explained as information transformation from one area to another (Stevenson et al., 2009; Yates et al., 2017; Perich et al., 2018; Rikhye et al., 2018; Hart and Huk, 2020). Conversely, shared latent variable models aim to elucidate interactions by identifying sources that capture shared fluctuations across areas (Keeley et al., 2020; Semedo et al., 2022). The ensemble GLM approaches and their integration with adaptive, Bayesian, or information-theoretic methods have also been able to capture functional connectivity or causal structures in inter- or intra-areal neural communications (Kim et al., 2011; Yates et al., 2017; Sheikhattar et al., 2018).

## 8 Discussion

Understanding how our behavior is generated through the integration of sensory, motor, and cognitive information relies on an ability to quantitatively describe the individual and interactive effects of external (e.g., visual input) and internal (e.g., attentional state) factors on neuronal responses. By capturing the statistical properties of neuronal responses with minimal assumptions about their functions, statistical models provide a powerful framework for describing the brain in terms of how it encodes information in its responses and decodes the resulting behavior. This is often difficult to achieve due to the complexities of neural data and limitations of computational models. In particular, our sensory processing and perception are dynamically modulated by many factors including context, attention, goals, reward, and learned associations. This will make the stimulus–response transformation the product of time-varying nonlinear interactions between sensory and non-sensory covariates. Understanding how these changes in the neuronal representation of sensory stimuli influence our perception of those stimuli and guide our behavior requires mathematically principled techniques that can track the information encoding and decoding in neuronal responses on the same timescale as the behavior.

Taking advantage of the richness and flexibility of the GLM framework, several studies have extended or generalized the classical GLM to incorporate more complicated factors beyond the sensory parameters, such as the neuron’s own spiking history, recent spiking of other cells, behavioral or task variables, and time-varying or state-dependent factors. These extended variants of GLMs allow for modeling more complicated response dynamics at the integration of sensory and non-sensory variables which is particularly a key feature of neurons in higher brain areas. Here we reviewed existing variations on GLMs and compared the common methods for dimensionality reduction and nonstationary responses. Lastly, we explored different applications of time-varying GLMs that showcased the value of these models for characterizing rapid neural dynamics in higher visual areas and investigating their role in creating various visual behaviors.

Our review summarized the different attempts in a point process GLM framework to characterize nonstationary aspects of spiking responses and discussed their advantages and limitations. These GLM-based paradigms are mostly based on white-noise-like stimuli, chosen for mathematical tractability in the fitting process, capturing unbiased kernels of the neurons, and generalizability to predict responses to unseen input patterns. However, previous studies suggest that fully characterizing neuronal responses requires naturalistic stimuli (Kayser et al., 2004; Nishimoto and Gallant, 2011; Talebi and Baker, 2012), and future work will need to extend such models to work on more realistic visual scenes. Nor do more complicated GLM-based paradigms scale in a tractable manner to capture high-dimensional structures such as the inter-neuronal relationships described by population-level models, or nonlinear and dynamic representation of stimulus space described by the modulatory or time-varying models. Although augmenting GLMs with regularization or dimensionality reduction procedures, such as sparse or low-dimensional regression models, have shown to be promising for tackling the dimensionality problem (Gerwinn et al., 2010; Sheikhattar et al., 2016; Aoi and Pillow, 2018; Zoltowski and Pillow, 2018; Niknam et al., 2019; Semedo et al., 2019), identifying behaviorally relevant dimensions (Akbarian et al., 2021; Sani et al., 2021; Valente et al., 2021; Weng et al., 2023) which can directly link representation to readout is another important direction for current and future research.

Beyond the variations of GLMs discussed here, there are numerous other extensions that have been developed. There are biologically-oriented modulatory models that incorporate the nonlinear effects of stimulus-driven suppression (Butts et al., 2007), or include contextual effects in sensory responses (Ahrens et al., 2008; Williamson et al., 2016), or implement a response gain control mechanism according to the stimulus contrast (Rabinowitz et al., 2011). The GLM-based approach has been used to provide mechanistic level understanding of several perceptual phenomena (Kayser et al., 2015; Zanos et al., 2016; Akbarian et al., 2021; Weng et al., 2023). A biophysical interpretation of one GLM has been suggested as a special case of a conductance-based encoding model, which bridged the disparity between statistical and biophysical models (Latimer et al., 2019).

The emergence of deep learning has introduced numerous novel methods for modeling neural responses. These methods can essentially be considered as an extended version of the GLM framework which allow for complex nonlinear response properties using their repetitive LN structures. It has been shown that artificial neural network (ANN) models can predict responses to natural stimuli in the retina (McIntosh et al., 2016) and in higher visual areas (Richards et al., 2019) with high accuracy, and many other network models have been established to examine neural circuits (Yamins et al., 2014; Moore et al., 2020). Despite some efforts in post-hoc interpretation of the network components (McIntosh et al., 2016) or incorporating behavioral variables into the network structure (Schneider et al., 2023), to date these neural network approaches have demonstrated limited generalizability or interpretability.

With the goal of incorporating the capabilities of biological neural networks such as temporal coding, spiking neural networks (SNNs) have been developed to mimic the properties of biological neurons, signal processing, and learning mechanisms (Maass, 1997). However, the existing SNNs currently trail ANNs in terms of accuracy, throughput, and energy efficiency (Khacef et al., 2018). Part of this gap can be attributed to inefficient architectural choices in SNNs such as imperfect signal encoding techniques, using rate-based codes, which lead to an inefficient way to perform communication and computation.

Statistical models such as GLM-like frameworks can characterize the functional link between neural response components and individual components of the neuron’s sensitivity and simultaneously provide statistical efficiency and computational tractability. As shown in this review, the GLM framework can be flexibly adapted to a multitude of tasks, experimental paradigms, types of neural data, temporal resolutions, circuit structures, choice of prior information, or feature selection procedures. Although this flexibility can be considered a powerful aspect of these models, their application-specific design may not be generally desirable, whereas the neural network models can allow for more general-purpose architectures and provide a better scalability. Taking advantage of the scalability of neural network encoders combined with large scale data such as two-photon or Neuropixels recordings, recent studies succeeded in extracting consistent and robust latent features across modalities, behaviors, or sessions (Schneider et al., 2023). On the other hand, neural network approaches may require a substantial volume of data, which can hinder their application in limited data conditions, such as in time-varying brain systems that play a crucial role in generating adaptive or flexible behaviors in real-time.

Taken together, GLM-like approaches based on regression and neural network-based approaches can be integrated in ways that inform and mutually enhance each other, allowing different applications to benefit from the desirable features of either approach. For example, understanding functional relationships between stimulus and single trial, single unit spiking responses or between multiple brain regions, obtained from regression-based models, may inform signal encoding strategies for enhancing SNNs to implement biologically plausible neuron models and communications in their computations and hardware. By combining the interpretability strength of regression-based models with flexible capabilities of VAE architectures, a recent study achieved better constrained and more identifiable latent embeddings (Zhou and Wei, 2020). Additionally, neural network models providing higher prediction accuracy can establish useful benchmarks for the performance of different GLM frameworks, or the design of GLM-based structures can inform a more data-efficient engineering of neural network models for different datasets.

Overall, the methods reviewed here, and future improvements building on these approaches, show great promise for revealing the computational and functional principles underlying neuronal processing of our dynamic and flexible behavior in a variety of brain functions and areas.

## Author contributions

GW: Conceptualization, Investigation, Validation, Writing – review & editing, Data curation, Formal analysis, Methodology, Software, Visualization, Writing – original draft. KC: Conceptualization, Visualization, Writing – review & editing. AA: Data curation, Investigation, Methodology, Software. BN: Writing – review & editing, Conceptualization, Funding acquisition, Project administration, Resources, Supervision, Validation. NN: Conceptualization, Funding acquisition, Project administration, Resources, Supervision, Validation, Writing – review & editing, Investigation.
